# Super-Resolution Imaging of Protein Secretion Systems and the Cell Surface of Gram-Negative Bacteria

**DOI:** 10.3389/fcimb.2017.00220

**Published:** 2017-05-29

**Authors:** Sachith D. Gunasinghe, Chaille T. Webb, Kirstin D. Elgass, Iain D. Hay, Trevor Lithgow

**Affiliations:** ^1^Infection and Immunity Program, Department of Microbiology, Biomedicine Discovery Institute, Monash UniversityClayton, VIC, Australia; ^2^Monash Micro Imaging, Monash UniversityClayton, VIC, Australia

**Keywords:** protein secretion, outer membrane, lipopolysaccharide, BAM complex

## Abstract

Gram-negative bacteria have a highly evolved cell wall with two membranes composed of complex arrays of integral and peripheral proteins, as well as phospholipids and glycolipids. In order to sense changes in, respond to, and exploit their environmental niches, bacteria rely on structures assembled into or onto the outer membrane. Protein secretion across the cell wall is a key process in virulence and other fundamental aspects of bacterial cell biology. The final stage of protein secretion in Gram-negative bacteria, translocation across the outer membrane, is energetically challenging so sophisticated nanomachines have evolved to meet this challenge. Advances in fluorescence microscopy now allow for the direct visualization of the protein secretion process, detailing the dynamics of (i) outer membrane biogenesis and the assembly of protein secretion systems into the outer membrane, (ii) the spatial distribution of these and other membrane proteins on the bacterial cell surface, and (iii) translocation of effector proteins, toxins and enzymes by these protein secretion systems. Here we review the frontier research imaging the process of secretion, particularly new studies that are applying various modes of super-resolution microscopy.

Fluorescence microscopy has proven to be a powerful tool for cell biologists, given the wide array of fluorescent probes available (fluorescent fusion proteins, reactive tags, and fluorescent antibodies) to specifically label and detect sub-cellular components in a cellular context. Together with increasingly higher quality optics, sensitive detectors and coherent light sources, the resolution capacity of fluorescence microscopy has now been extended to generate superior images with finer details than ever before. Until these recent developments, microbiologists were unable to fully capitalize on fluorescence microscopy, since the diffraction limit of light means only objects larger than ~250 nm in lateral dimension and ~500 nm in axial dimension could be resolved: any objects smaller than these limits are merely blurred spots (Patterson et al., 2010). Many of the structures of interest in microbes are much smaller than this classical limit, with bacteria themselves only 1–10 μm in length (Koch, 1996).

The advent of super-resolution microscopy extended the classical limit imposed by conventional light microscopy (Hell, 2007, 2009; Huang et al., 2009, 2010). There are two general classes of super-resolution microscopy. The first class of imaging modalities utilizes spatially patterned fluorescence excitation beams to achieve the sub-diffraction level of resolution. The most notable examples of this technique are stimulated emission depletion (STED) microscopy (Hell and Wichmann, 1994; Klar and Hell, 1999), reversible saturable optical fluorescence transitions (RESOLFT) microscopy (Hell and Wichmann, 1994; Hofmann et al., 2005) and structured illumination microscopy (SIM) (Gustafsson, 2000, 2005). The second class circumvents the diffraction barrier through actively controlling the fluorescence emitter (fluorescent proteins, antibodies or tags) concentrations by stochastic photo-activation or by stochastic photo-switching (Heilemann et al., 2009; Lippincott-Schwartz and Patterson, 2009; Kamiyama and Huang, 2012), thereby enabling spatio-temporal resolution of emitter localizations. This class includes photoactivation localization microscopy (PALM) (Betzig et al., 2006) and stochastic optical reconstruction microscopy (STORM), collectively known as single molecule localization microscopy (SMLM) (Rust et al., 2006). These techniques are documented to reach 10–25 nm of lateral resolution (Kamiyama and Huang, 2012), a scale that allows visualization of macromolecules in small cellular systems (Figure 1).

**Figure 1:**
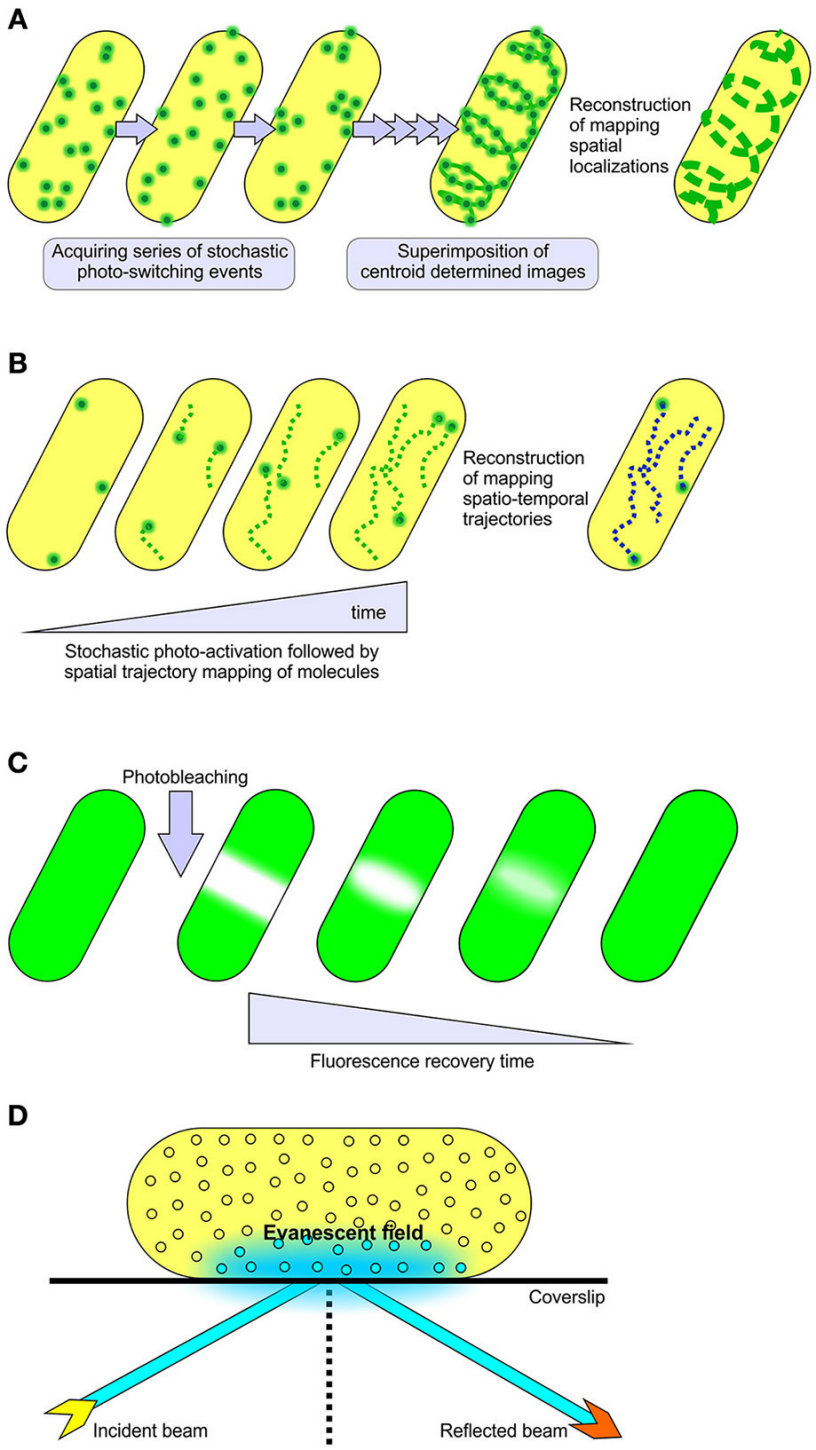
Methods for single molecule localization and tracking. **(A)** STORM and **(B)** PALM super-resolution microscopy, collectively referred to as single molecule localization microscopy methods (SMLM). These modalities utilize stochastic photo-switching or photo-activation of a subset of fluorescence emitters to achieve sub-diffraction resolution of 10–25 nm in lateral dimensions. PALM is well known for live cell imaging and mapping spatiotemporal trajectories of individual fluorescently labeled molecules (Manley et al., 2008). STORM is documented for its use in determining the localized positioning of fluorescently labeled molecules in both live and fixed samples (Kamiyama and Huang, 2012). The mobility of fluorescent fusion proteins in live cells can also be surveyed through **(C)** FRAP and **(D)** TIRF microscopy techniques. In FRAP experiments, a relatively small area within the cell is irreversibly photo-bleached by a high intensity laser beam, followed by subsequent monitoring of the redistribution of non-bleached fluorescence molecules into the photo-bleached region under low laser power. TIRFM relies upon the generation of a rapidly decaying evanescent field at the interface of sample and coverslip to sparsely excite fluorophores, providing a high signal to background noise ratio in single particle tracking studies (Toomre and Bewersdorf, 2010).

In order to exploit their environmental niches, bacteria undertake vital tasks such as sensing the external milieu, cell to cell communication, nutrient uptake against concentration gradients, cell-cell warfare and the secretion of macromolecules into the environment. Gram-negative bacteria have a highly evolved cell wall with two membranes composed of a complex array of integral and peripheral proteins, as well as phospholipids and glycolipids. The outer membrane is an asymmetric bilayer, with an inner leaflet of phospholipids and an outer leaflet of lipopolysaccharide (LPS). As discussed herein, we are beginning to appreciate that this asymmetric lipid environment promotes spatial heterogeneity of membrane constituents and impedes the sort of lateral mobility that is common for the proteins integrated in phospholipid bilayers. Super resolution microscopy is being applied to dissect diverse aspects of bacterial cell biology, including membrane protein structure and dynamics (Xie et al., 2008). In this review, we highlight the advances that have been made in understanding spatial-temporal characteristics of bacterial surface proteins, particularly protein secretion systems, that the recent advances in microscopy have allowed. To date, model bacterial systems like *Escherichia coli* and *Caulobacter cresentus* have been the subject for the majority of single molecule localization studies (Gahlmann and Moerner, 2014).

## Fluorescence imaging of outer membrane structure and biogenesis

Most bacterial outer membrane proteins (OMPs) have a β-barrel architecture (De Geyter et al., 2016; Plummer and Fleming, 2016; Noinaj et al., 2017; Slusky, 2017) and, of these, the channels that allow for selective permeability of small molecules across the outer membrane are referred to as porins (Hancock, 1987). Some of these porins display surface exposed extracellular domains, often simply loops of polypeptide between adjacent β-strands, which none the less provide the means to fluorescently label them for mobility assessment studies on live cells (Gibbs et al., 2004; Spector et al., 2010; Rassam et al., 2015). With the aid of fluorescent recovery after photo-bleaching (FRAP) and single particle tracking using total internal reflection fluorescence microscopy (TIRFM), we are now beginning to understand time-resolved spatial movements of these outer membrane proteins (Gibbs et al., 2004; Spector et al., 2010; Rothenberg et al., 2011; Rassam et al., 2015).

LamB is a trimeric porin responsible for maltose uptake in *E. coli* (Schirmer et al., 1995). LamB also serves as the receptor for certain bacteriophage (Chatterjee and Rothenberg, 2012), and has been extensively studied in terms of diffusion dynamics. Using various labeling techniques and different imaging modalities, LamB mobility has been described using parametric measurements such as the short-time diffusion coefficient. In essence, this quantifies the area a molecule inhabits in a per second measurement. LamB displays a short-time diffusion coefficient of 0.15 – 0.06 μm^2^s^−1^, with each molecule therefore being confined to a space of ~20 nm at the outer membrane (Oddershede et al., 2002; Gibbs et al., 2004; Rothenberg et al., 2011). Similar results have come from study of other porins. OmpF, for example, was reported to have short-time diffusion coefficients of 0.006 μm^2^s^−1^ (Spector et al., 2010). Similarly, a short-time diffusion coefficient of 0.05 μm^2^s^−1^ was reported for the TonB-dependent receptor BtuB, which facilitates cobalamin uptake (Spector et al., 2010). For both OmpF and BtuB, recent work has suggested that their distribution and relative immobility may be due to non-specific, protein-protein interactions (Rassam et al., 2015). OmpA functions to lock the outer membrane to the under-lying peptidoglycan layer, and it had been expected that this feature alone would dictate the relative immobility predicted for OmpA (Samsudin et al., 2016). However, deletion of peptidoglycan binding domain of OmpA, did not affect the diffusion coefficient measurements for the β-barrel domain of OmpA (Verhoeven et al., 2013). These and other studies have led to the understanding that, compared to inner membrane proteins, OMPs generally display orders of magnitude slower diffusion dynamics whether or not they are tethered to other cellular structures (Oddershede et al., 2002; Gibbs et al., 2004; Spector et al., 2010; Rothenberg et al., 2011; Ritchie et al., 2013; Verhoeven et al., 2013; Rassam et al., 2015).

In this emerging paradigm of membrane spatial rigidity, it has become clear that the distribution of LPS is also greatly constrained. By fluorescently labeling LPS via the α-mannose moiety of its O-antigen, distinct helical ribbon-like geometric arrangements were observed for LPS on live *E.coli* (Ghosh and Young, 2005). Very low diffusion coefficients reported by FRAP experiments showed that LPS molecules were practically immobile by comparison with the (already very low) OMP diffusion rates (Mühlradt et al., 1973; Schindler et al., 1980). The current hypothesis is that LPS helical ribbons may represent a geometric arrangement important for staging outer membrane biogenesis. Given the high abundance yet constrained spatial distribution of LPS, it is becoming clear that any model for protein transport into or across the outer membrane will need to take into account this spatial information.

Few studies have yet to directly address the spatio-temporal aspects of the process of β-barrel assembly into the outer membrane. In one, temporal labeling of LamB appearance on the bacterial cell surface has been studied in elegant work, using detailed computational analysis to reconstruct the first spatio-temporal distribution of OMP biogenesis (Ursell et al., 2012). Employing site specific protein labeling strategy using Sfp phosphopantetheinyl transferase to covalently label emergent loops of nascent LamB molecules, the appearance and mobility of LamB molecules was monitored through time-lapse fluorescence microscopy. Inducible pulse-chase expression of LamB revealed an initial emergence of fluorescent punctae which represents a heterogeneous distribution of fluorescent spots per bacterium. The heterogeneous localization of LamB was due to discrete bursts of insertion of new material at discrete sites throughout the outer membrane. This presumably relates to the number and location of active β-barrel assembly machinery (BAM) complexes, which serve to catalyse β-barrel protein assembly into the outer membrane (De Geyter et al., 2016; Plummer and Fleming, 2016; Noinaj et al., 2017). The numbers of these punctae is similar to the number of sites estimated in an early EM-based study that captured porin insertion sites in *Salmonella* Typhimurium using ferritin-conjugated OMP-specific antibodies (Smit and Nikaido, 1978). Importantly, FRAP experiments showed that any laterally measurable movement of the OMPs across the bacterial cell surface was dependent on membrane growth and was not diffusional (Ursell et al., 2012). Since LamB also serves as receptor for several bacteriophage (Hancock and Reeves, 1976), fluorescently labeled λ phage tails have also been used to monitor the endogenous distribution of LamB—without plasmid-borne over-expression—and these studies too find it to be driven by cell growth and elongation (Gibbs et al., 2004).

The process of outer membrane biogenesis also depends on OMP turn-over through generational change in an *E. coli* population. A recent study using covalently modified colicins to fluorescently label two OMPs, BtuB and Cir, elegantly followed this process through TIRFM (Rassam et al., 2015). BtuB and Cir were observed to be clustered together in “OMP islands,” huge rafts with an average size of ~ 0.5 μm (hundreds to thousands of proteins molecules would be encompassed in this island, with little or no interstitial lipid present). Unlike the LamB studies, where new material was delivered at points all across the cell surface, Rassam et al. suggested that the insertion of new BtuB and Cir into these rafts was only observed in the mid-cell region (Rassam et al., 2015). Irrespective of the site of new material deposition, computer modeling studies (Wang et al., 2008; Ursell et al., 2012; Rassam et al., 2015) have demonstrated that in either scenario, pre-existent OMPs and LPS will always tend to be forced toward poles, and that cell division will ultimately yield an unequal partitioning of membrane materials to create distinct subpopulations of cells, ones having mixed set of old and new material and others with predominantly or exclusively “young” OMPs. Within a bacterial population this then creates a range of phenotypes in the outer membrane proteome, and a range of adaptive advantages for individual bacteria to survive and replicate in that environment. In various ways, other studies have demonstrated how “older” elements of the other major cell envelope constituents, LPS and peptidoglycan, are also ultimately retained at cell poles (Kato et al., 1990, 2000; De Pedro et al., 2003; Thiem et al., 2007; Thiem and Sourjik, 2008). *In silico* models have predicted this type of protein clustering and binary partitioning of membrane proteins. For example, this temporal positioning has been observed to be important in resolving protein aggregates associated with bacterial cell aging, but is also important in positioning chemoreceptor arrays and regulation of cell division (Janakiraman and Goldberg, 2004; Thiem et al., 2007; Lindner et al., 2008; Thiem and Sourjik, 2008).

## Fluorescence-based imaging of bacterial secretion systems

**Figure 2:**
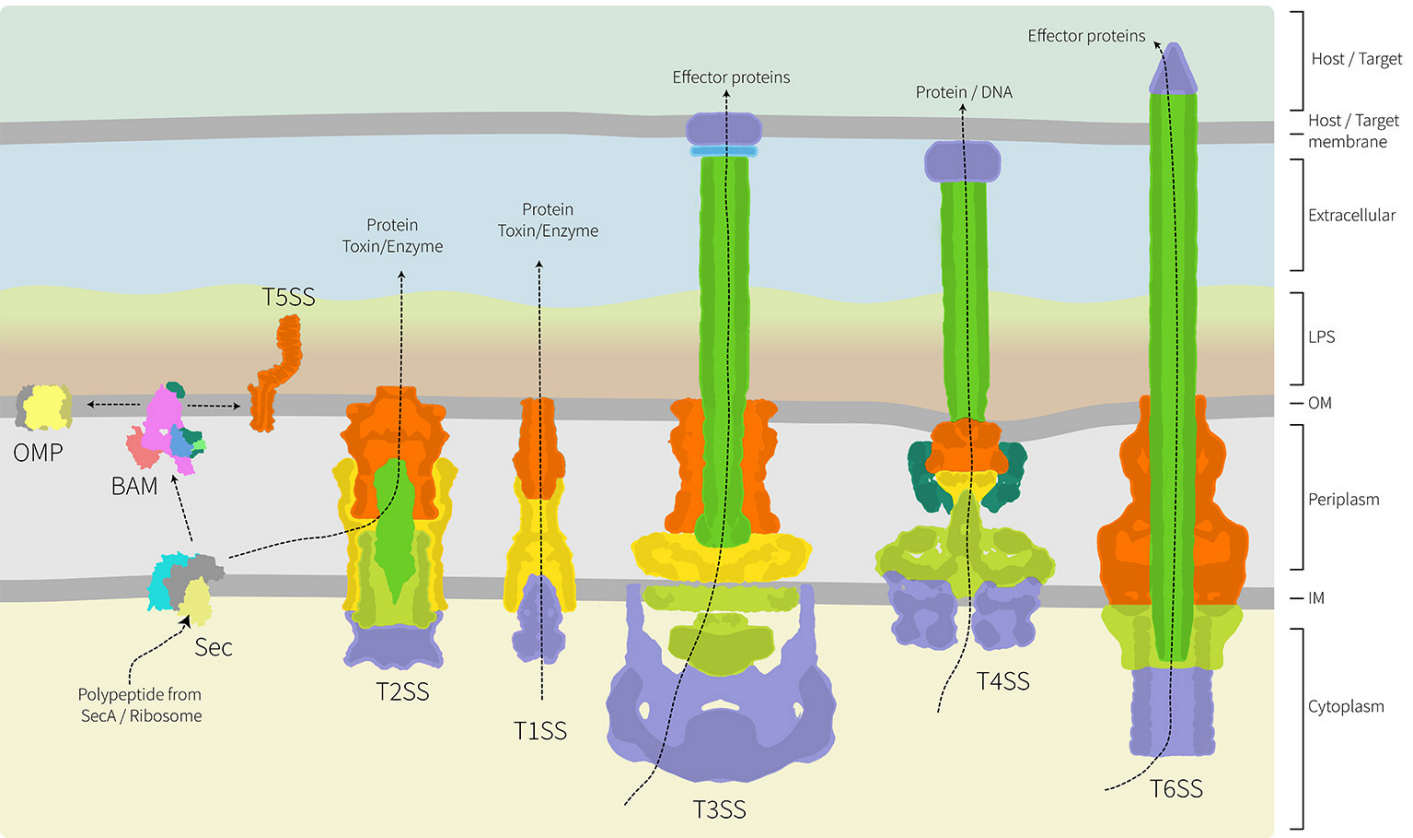
Architecture of the major protein secretion systems found in Gram-negative bacteria. Of the six major protein secretion systems, five span the inner and outer membranes and the periplasm. These nanomachines are thereby trapped in the peptidoglycan layer. The T1SS, T3SS, T4SS, and T6SS collect substrate proteins directly from the cytoplasm for secretion across both membranes, while the T2SS collects substrate proteins from the periplasm with their delivery there being via the Sec translocon (shown) or Tat transport system (not shown). While informed artistic license was taken with the placement, orientation and shape of some proteins/complexes, wherever possible relative structural information was used. The structural data used as a basis in the preparation of this figure are: PDB 1ek9, 5l22, 5mg3, 5d0o, 1mal, 5ksr, 5wq8, 3fpp, 4ksr, 5l22, 3fpp, 4kh3, 4mee; and EMD-2927, EMD-2667, EMD-2567, EMD-1875. The structure and function of these various protein translocation systems are detailed elsewhere in this special volume.

A frontier area of research in bacterial cell biology concerns the assembly and distribution of protein secretion machines. Bacteria have evolved numerous mechanisms to efficiently secrete proteins into the environment and characterization of several protein secretion systems is yielding exciting advances in understanding their structure and function (Dalbey and Kuhn, 2012; Costa et al., 2015). Because these systems are detailed elsewhere in this special edition, we have focused our review on studies where spatial distribution appears to be important either to the biogenesis of the secretion system, or to its function. As highlighted in Figure 2, the architecture of these secretion nanomachines can differ from single component systems, to relatively simple systems composed of only 3 subunits, to multicomponent systems containing over 20 protein subunits and spanning all four compartments of the bacterial cell (Dalbey and Kuhn, 2012; Campos et al., 2013; Galán et al., 2014; Ho et al., 2014; Thomas et al., 2014; Trokter et al., 2014; van Ulsen et al., 2014; Zoued et al., 2014; Basler, 2015; Costa et al., 2015; Fan et al., 2016; Notti and Stebbins, 2016).

## Type 1 secretion system (T1SS)

Effector proteins secreted through T1SS have been characterized in a number of bacterial pathogens including in uropathogenic *E. coli, Bordetella pertussis* and more recently through effector protein RtxA in *Legionella pneumophila* (Brooks et al., 1980; Shrivastava and Miller, 2009; Fuche et al., 2015). Using deletion mutants and fluorescence protein fusion constructs of RtxA (photoactivatable mCherry-RtxA), secretion was monitored through the course of infection. *Legionella* were internalized by host cells into the endoplasmic reticulum-like compartment, the *Legionella*-containing vacuole, where bacterial replication takes place inside the host. With the versatility of genetically-encoded, reactive tags (Halo-tag and SNAP-tag), Barlag et al. imaged subunits of T1SS in *Salmonella enterica* at a nanoscopic level (Barlag et al., 2016). Diffusion coefficients reported for SiiF, the inner membrane component the T1SS were 0.008 μm^2^s^−1^ and the secretion machine was confined into 3–4 localized punctae. This very low diffusion coefficient for an inner membrane protein is perhaps predictable, given that T1SS include a TolC-type protein integrated into the outer membrane (Thomas et al., 2014), and in consideration that by spanning the periplasm the T1SS would be trapped within the peptidoglycan meshwork. This peptidoglycan trap would likely apply equally in constraining the movement of other protein secretion systems too (Figure 2). Together, these studies illustrate the resourcefulness of fluorescent fusion and enzyme tags and how these can be utilized in super resolution imaging of the secretion machines and their substrates, without altering the functionality of either.

## Type 2 secretion system (T2SS)

The T2SS has been identified in a number of bacterial pathogens, and is particularly well known in *Vibrio cholerae* as the system responsible for cholera toxin secretion (Sandkvist et al., 1997; Connell et al., 1998; Lybarger et al., 2009; Sikora et al., 2011). Elements required for the assembly of this T2SS are encoded by the extracellular protein secretion (*eps*) genes (Overbye et al., 1993; Sandkvist et al., 1997; Marsh and Taylor, 1998; Fullner and Mekalanos, 1999) and the localization of fluorescently labeled protein subunits in *V. cholerae* has revealed insight into the assembly and spatial dynamics. Using chromosomal and plasmid borne GFP fusions with the EpsM, EspG and EpsC subunits of the T2SS the subcellular localization of each components was assessed in *Vibrio cholerae* (Lybarger et al., 2009). When chromosomally-expressed (to produce endogenous steady-state levels of protein), GFP-EpsM formed distinct fluorescent foci that were not concentrated at cell poles. Conversely, when plasmid-borne over-expression of GFP-EpsM was instituted, the protein displayed a polar localization.

This discernible effect on protein subcellular localization under differential gene expression is a concern that needs careful consideration. The impact of overexpression on physiologically-relevant vs. non-physiological localizations is equally of concern in studies on other protein secretion systems (and all subcellular structures) too. As mentioned previously, protein aggregates tend to be deposited in polar locations (Janakiraman and Goldberg, 2004; Thiem et al., 2007; Lindner et al., 2008; Thiem and Sourjik, 2008).

Type 4 pili are a locomotional appendage that are ancestrally and structurally related to the T2SS (Peabody et al., 2003). The distribution of T4 pili has been followed using fluorescent protein fusions. An mCherry fusion tag was attached to the BfpB subunit in the Type 4 pili of enteropathogenic *E. coli* (Lieberman et al., 2012). PALM was used to capture single molecule localization events of BfpB-PAmCherry, providing evidence for a largely non-polar distribution pattern in Type 4 pili biogenesis, as is likely in T2SS biogenesis. Conversely, studies in *Pseudomonas aeruginosa* suggest that Type 4 pili biogenesis occurs at sites of cell division: that is, that the nanomachines are pre-installed at what will become a cell pole (after cell division concludes) (Carter et al., 2017). Taken together these exciting studies highlight the complex spatio-temporal scenarios that exist in the cell biology of bacteria, and caution against concluding that what is true in *E. coli* is necessarily true in other bacterial species.

## Type 3 secretion system (T3SS)

The T3SS has been identified in several bacterial pathogens including *Yersinia enterocolitica, E. coli, Shigella flexneri*, and *Salmonella* Typhimurium. While electron microscopy and electron tomography have provided exquisite detail in the structure of these complicated nanomachines (Spreter et al., 2009; Carleton et al., 2013; Radics et al., 2014; Hu et al., 2015, 2017), a number of different fluorescence microscopy imaging studies on live cells have provided in-depth analysis on different stages of T3SS assembly and effector protein secretion into host cells. Time-lapse fluorescence microscopy has monitored the T3SS during *S*. Typhimurium infection of epithelial cells (Schlumberger et al., 2005). A fluorescently-labeled substrate of the T3SS, SipA, was monitored for its emergence into the host cells, and its concomitant depletion from the bacterial cytoplasm. At the event of infection, it was estimated via fluorescence measurements that *S*. Typhimurium was able to deliver 6 ± 3 × 10^3^ molecules of SipA to the host cell within 100–600 s, and the effector protein ejection starts ~16–25 s after docking on to the host. In terms of the nanomachine itself, subunits of the T3SS in *S*. Typhimurium have been visualized too (Diepold et al., 2010, 2015; Kudryashev et al., 2015; Notti et al., 2015; Barlag et al., 2016). The diffusion coefficient for SpaS, an inner membrane component of the T3SS, was measured to be 0.055 μm^2^s^−1^, which is a similar scale to the diffusion coefficients measured for outer membrane proteins LamB and OmpF. While not directly tested, this level of immobility of the inner membrane elements of the T3SS is consistent with them being constrained in their diffusional movements by the outer membrane components of the nanomachine and the peptidoglycan trap. The same would be true of the T1SS (Barlag et al., 2016), where SiiF is found in the inner membrane but would be constrained in its diffusional movement by its spanning the peptidoglycan and attached to the TolC-homolog SiiC in the outer membrane (Kiss et al., 2007). In these studies, the localization of the T3SS was distributed relatively evenly across the bacterial cell. It would be of great interest to monitor the T3SS distribution in pathogens such as enteropathogenic *E. coli* during the initial events of host cell encounter, when only a single surface of the bacterium engages the epithelial cell surface to initiate pathogen attachment and effacement (Wong et al., 2011; Gaytán et al., 2016). Is there a random engagement of only some T3SS with the host cell? Or does the pathogen control the spatial arrangement of T3SS to ensure maximal engagement on the “host side” of its surface?

## Type 4 secretion system (T4SS)

The T4SS functions to transfer proteins and/or DNA in bacterial conjugation encounters or during infection (Ding et al., 2003; Trokter et al., 2014). The Dot/Icm machinery is the T4SS in *Legionella* spp. and is responsible for hundreds of effector proteins throughout the infection cycle (So et al., 2015). Epitope-tagging one of these effectors, LncP, enabled fluorescence imaging of infected macrophages and showed that the protein was secreted from the bacterial cytoplasm by the T4SS and ultimately translocated across a remarkable five membranes in order to be assembled into the mitochondrial inner membrane of the host cell (Dolezal et al., 2012). An equivalent T4SS is found in *Coxiella burnetii*, and has been reported to be exclusively located at one pole of the bacterium (Morgan et al., 2010). It has been suggested that in these species, bacteria-host membrane contact might be required to initiate secretion into the host cell cytoplasm (Voth and Heinzen, 2007). That being the case, a unique localization of the T4SS nanomachines would promote the efficiency of effector protein secretion.

The T4SS has been well characterized in the plant pathogen *Agrobacterium tumefaciens* (Zupan et al., 2000), where it is composed of 12 subunits (Das and Xie, 2000; Ward et al., 2002; Jakubowski et al., 2003; Low et al., 2014; Trokter et al., 2014; Chandran Darbari and Waksman, 2015; Costa et al., 2015) and facilitates the delivery of tumor inducing pTi plasmid to plant cells, causing overproduction of certain plant growth hormones and leading to a resultant tumor (Dessaux et al., 1993). Initial spatial location studies on the T4SS in *A. tumefaciens* suggested that the VirD4 and VirB6 components were located at cell poles (Kumar and Das, 2002; Judd et al., 2005). Using deconvolution fluorescence microscopy and antibodies to specifically label the T4SS in *A. tumefaciens*, multiple clustered regions were observed along the cell periphery, i.e., not at the cell poles, in a majority of bacterial cells (Aguilar et al., 2011). Based on these finding, the authors proposed a lateral attachment model providing a more effective means for contact between pathogen and host during *A. tumefaciens* infection compared to a cell pole mediated attachment.

## Type 5 secretion system (T5SS)

There are several sub-types of T5SS, including autotransporters, inverse autotransporters, two-partner secretion systems and others (Fan et al., 2016; Heinz et al., 2016). Fluorescence microscopy studies on autotransporters derived from a variety of bacteria, including AIDA-I from *E. coli*, IcsA and SepA from *Shigella flexneri*, BrkA from *Bordetella pertussis*, and BimA from *Burkholderia thailandensis*, were shown to be directly localized to bacterial cell poles when translocated to the cell surface (Charles et al., 2001; Jain et al., 2006; Lu et al., 2015). Indeed, when IcsA, SepA, and BrkA were expressed in *E. coli* systems, they still migrated to the poles, suggesting that intrinsic features in the autotransporters programmes this polar localization (Jain et al., 2006). Conversely, at least one autotransporter, Ag43, was localized as covering the whole cell surface without any concentration toward poles (Danese et al., 2000; Kjærgaard et al., 2000). It is important to note that none of these studies have addressed where the integration event took place, nor any dynamics of movement of the autotransporters, but rather visualized their steady-state positioning. In addition to the effects of cell division or LPS leading to polar localization, the location at which a protein is translocated across the cytoplasmic membrane, through processes collectively considered as “transertion” (Bakshi et al., 2014; Matsumoto et al., 2015), may be important in determining how a protein achieves polar localization.

## Type 6 secretion system (T6SS)

While being the most recently discovered of the major protein secretion systems in Gram-negative bacteria, predictions suggest the T6SS to be present in ~25% of species (Basler et al., 2012). Characterization studies show that the T6SS functions in virulence for several pathogens including *B. thailandensis, Pseudomonas aeruginosa, Serratia marcescens* and *V. cholerae* (Mougous et al., 2006; Pukatzki et al., 2006, 2007; Schwarz et al., 2014). Structural elucidation of the T6SS from *V. cholerae* revealed startling similarities to bacteriophage tails. In general, T6SSs are assembled to contain a contractible sheath, baseplate and a membrane puncturing spike to mediate effector protein secretion into host cells (Basler et al., 2012; Filloux, 2013). Using a super-folder green fluorescent protein (sfGFP) fusion to VipA, one of the two protein components that makeup the contractile sheath, long straight tubular structures were localized in the cytoplasm extending along the width or length of the bacterium (Basler et al., 2012). Time-lapse fluorescence microscopy revealed these sub-cellular structures were highly dynamic in nature. They assemble at 20–30 s μm^−1^, rapidly contracted to about 50% from their original length within ≤5 ms, and finally disassembled over a 30–60 s period (Basler et al., 2012). Recent studies using a similar fluorescent protein fusion tag approach captured the baseplate protein TssA joining the sheath component's polymerization in *E. coli* (Zoued et al., 2016).

T6SSs are often used in inter-species warfare, in order to outcompete bacterial neighbors (Hood et al., 2010; MacIntyre et al., 2010; Schwarz et al., 2010; Murdoch et al., 2011; Zoued et al., 2014; Journet and Cascales, 2016). Time-lapse fluorescence microscopy quantitatively demonstrated this antibacterial activity through predator-prey cell dynamics on live cells (Brunet et al., 2013). Co-culturing a pathogenic, “predator” *E. coli* strain expressing TssB-sfGFP (TssB is alternatively known as VipA) and a non-pathogenic, “prey” *E. coli* strain devoid of T6SS expressing fluorescent protein mCherry, showed that only upon contact with the prey was the contraction of the sheath structure triggered in predator cells. Conversely, in *S. marcescens* equivalent experimental strategies showed that the bacteria does not wait to encounter prey (or enemy) cells, but behaves aggressively and fires the T6SS irrespective of any provocation by cell-cell contact (Gerc et al., 2015). These studies again highlight the diverse behaviors that different bacterial species have evolved to deploy against their neighbors and enemies, and provide fascinating insight into the dynamics of how T6SS effector proteins are secreted to target other bacteria.

## Conclusion

It is early in the application of super resolution microscopy techniques to capture the cellular events in the biogenesis and action of protein secretion systems in bacteria. Already, studies have challenged our preconceptions on bacterial cell envelope organization and protein dynamics. For example, the outer membrane is not a fluid mosaic, but a turgid structure that constrains membrane protein movement. Selective deployment of a protein secretion system is thereby possible at highly precise locations. High resolution imaging modalities will be tremendously useful in answering long-standing questions in bacterial cell biology, such as what mechanisms drive the biogenesis of outer membrane vesicles (Turnbull et al., 2016), questions which until recently have largely been experimentally intractable. A further example is the extent to which transertion—the coupling of transcription, translation, and translocation—provides a mechanism for highly localized distributions of membrane proteins. This review has indicated the uses for the growing number of versatile fluorescent probes, which can be used in the distinct cellular environments, and how they promise to extend fluorescence microscopy applications even further. Examining host-pathogen interactions, detailing the nanoscale organization of protein secretion systems and studying the dynamic nature of sub-cellular compartments in live bacteria is now possible through super resolution microscopy. In the future, no doubt, these imaging techniques will be applied to understand the cell biology of the grand diversity of bacterial species, beyond characteristic model bacteria.

## Author contributions

SG read the literature and wrote the manuscript. CW read the literature and wrote the manuscript. KE read the literature and wrote the manuscript. IH read the literature and wrote the manuscript. TL read the literature and wrote the manuscript.

### Conflict of interest statement

The authors declare that the research was conducted in the absence of any commercial or financial relationships that could be construed as a potential conflict of interest.

## References

[B1] Aguilar J. Cameron T. A. Zupan J. Zambryski P. (2011). Membrane and core periplasmic *Agrobacterium tumefaciens* virulence Type IV secretion system components localize to multiple sites around the bacterial perimeter during lateral attachment to plant cells. MBio 2, e00218–e00211. 10.1128/mBio.00218-11 22027007 PMC3202754

[B2] Bakshi S. Choi H. Mondal J. Weisshaar J. C. (2014). Time-dependent effects of transcription- and translation-halting drugs on the spatial distributions of the *Escherichia coli* chromosome and ribosomes. Mol. Microbiol. 94, 871–887. 10.1111/mmi.12805 25250841 PMC4227943

[B3] Barlag B. Beutel O. Janning D. Czarniak F. Richter C. P. Kommnick C.. (2016). Single molecule super-resolution imaging of proteins in living *Salmonella enterica* using self-labelling enzymes. Sci. Rep. 6:31601. 10.1038/srep31601 27534893 PMC4989173

[B4] Basler M. Pilhofer M. Henderson G. P. Jensen G. J. Mekalanos J. J. (2012). Type VI secretion requires a dynamic contractile phage tail-like structure. Nature 483, 182–186. 10.1038/nature10846 22367545 PMC3527127

[B5] Basler M. (2015). Type VI secretion system: secretion by a contractile nanomachine. Philos. Trans. R. Soc. B 370:20150021. 10.1098/rstb.2015.0021 26370934 PMC4632598

[B6] Betzig E. Patterson G. H. Sougrat R. Lindwasser O. W. Olenych S. Bonifacino J. S.. (2006). Imaging intracellular fluorescent proteins at nanometer resolution. Science 313, 1642–1645. 10.1126/science.1127344 16902090

[B7] Brooks H. J. O'Grady F. McSherry M. A. Cattell W. (1980). Uropathogenic properties of *Escherichia coli* in recurrent urinary-tract infection. J. Med. Microbiol. 13, 57–68. 10.1099/00222615-13-1-57 6102156

[B8] Brunet Y. R. Espinosa L. Harchouni S. Mignot T. Cascales E. (2013). Imaging type VI secretion-mediated bacterial killing. Cell Rep. 3, 36–41. 10.1016/j.celrep.2012.11.027 23291094

[B9] Campos M. Cisneros D. A. Nivaskumar M. Francetic O. (2013). The type II secretion system–a dynamic fiber assembly nanomachine. Res. Microbiol. 164, 545–555. 10.1016/j.resmic.2013.03.013 23542426

[B10] Carleton H. A. Lara-Tejero M. Liu X. Galan J. E. (2013). Engineering the type III secretion system in non-replicating bacterial minicells for antigen delivery. Nat. Commun. 4:1590. 10.1038/ncomms2594 23481398 PMC3693737

[B11] Carter T. Buensuceso R. N. Tammam S. Lamers R. P. Harvey H. Howell P. L.. (2017). The type IVa pilus machinery is recruited to sites of future cell division. MBio 8:e02103–16. 10.1128/mBio.0210316 28143978 PMC5285504

[B12] Chandran Darbari V. Waksman G. (2015). Structural biology of bacterial type IV secretion systems. Annu. Rev. Biochem. 84, 603–629. 10.1146/annurev-biochem-062911-102821 26034891

[B13] Charles M. Pérez M. Kobil J. H. Goldberg M. B. (2001). Polar targeting of *Shigella* virulence factor IcsA in *Enterobacteriacae* and *Vibrio*. Proc. Natl. Acad. Sci. U.S.A. 98, 9871–9876. 10.1073/pnas.171310498 11481451 PMC55545

[B14] Chatterjee S. Rothenberg E. (2012). Interaction of bacteriophage λ with its *E. coli* receptor, LamB. Viruses 4, 3162–3178. 10.3390/v4113162 23202520 PMC3509688

[B15] Connell T. D. Metzger D. J. Lynch J. Folster J. P. (1998). Endochitinase is transported to the extracellular milieu by the eps-encoded general secretory pathway of *Vibrio cholerae*. J. Bacteriol. 180, 5591–5600. 9791107 10.1128/jb.180.21.5591-5600.1998 PMC107616

[B16] Costa T. R. Felisberto-Rodrigues C. Meir A. Prevost M. S. Redzej A. Trokter M.. (2015). Secretion systems in Gram-negative bacteria: structural and mechanistic insights. Nat. Rev. Microbiol. 13, 343–359. 10.1038/nrmicro3456 25978706

[B17] Dalbey R. E. Kuhn A. (2012). Protein traffic in Gram-negative bacteria–how exported and secreted proteins find their way. FEMS Microbiol. Rev. 36, 1023–1045. 10.1111/j.1574-6976.2012.00327.x 22250915

[B18] Danese P. N. Pratt L. A. Dove S. L. Kolter R. (2000). The outer membrane protein, Antigen 43, mediates cell-to-cell interactions within *Escherichia coli* biofilms. Mol. Microbiol. 37, 424–432. 10.1046/j.1365-2958.2000.02008.x 10931336

[B19] Das A. Xie Y. H. (2000). The *Agrobacterium* T-DNA transport pore proteins VirB8, VirB9, and VirB10 interact with one another. J. Bacteriol. 182, 758–763. 10.1128/JB.182.3.758-763.2000 10633111 PMC94340

[B20] De Geyter J. Tsirigotaki A. Orfanoudaki G. Zorzini V. Economou A. Karamanou S. (2016). Protein folding in the cell envelope of *Escherichia coli*. Nat. Microbiol. 1:16107. 10.1038/nmicrobiol.2016.107 27573113

[B21] De Pedro M. A. Schwarz H. Koch A. L. (2003). Patchiness of murein insertion into the sidewall of *Escherichia coli*. Microbiology 149, 1753–1761. 10.1099/mic.0.26125-0 12855727

[B22] Dessaux Y. Petit A. Tempe J. (1993). Chemistry and biochemistry of opines, chemical mediators of parasitism. Phytochemistry 34, 31–38. 10.1016/S0031-9422(00)90778-7

[B23] Diepold A. Amstutz M. Abel S. Sorg I. Jenal U. Cornelis G. R. (2010). Deciphering the assembly of the *Yersinia type* III secretion injectisome. EMBO J. 29, 1928–1940. 10.1038/emboj.2010.84 20453832 PMC2885934

[B24] Diepold A. Kudryashev M. Delalez N. J. Berry R. M. Armitage J. P. (2015). Composition, formation, and regulation of the cytosolic c-ring, a dynamic component of the type III secretion injectisome. PLoS Biol. 13:e1002039. 10.1371/journal.pbio.1002039 25591178 PMC4295842

[B25] Ding Z. Atmakuri K. Christie P. J. (2003). The outs and ins of bacterial type IV secretion substrates. Trends Microbiol. 11, 527–535. 10.1016/j.tim.2003.09.004 14607070 PMC4844353

[B26] Dolezal P. Aili M. Tong J. Jiang J. H. Marobbio C. M. Lee S. F.. (2012). *Legionella pneumophila* secretes a mitochondrial carrier protein during infection. PLoS Pathog. 8:e1002459. 10.1371/journal.ppat.1002459 22241989 PMC3252375

[B27] Fan E. Chauhan N. Udatha D. G. Leo J. C. Linke D. (2016). Type V secretion systems in bacteria. Microbiol. Spectr. 4:VMBF-0009-2015. 10.1128/microbiolspec.VMBF-0009-2015 26999388

[B28] Filloux A. (2013). Microbiology: a weapon for bacterial warfare. Nature 500, 284–285. 10.1038/nature12545 23925115

[B29] Fuche F. Vianney A. Andrea C. Doublet P. Gilbert C. (2015). Functional type 1 secretion system involved in *Legionella pneumophila* virulence. J. Bacteriol. 197, 563–571. 10.1128/JB.02164-14 25422301 PMC4285970

[B30] Fullner K. J. Mekalanos J. J. (1999). Genetic characterization of a new type IV-A pilus gene cluster found in both classical and El Tor biotypes of *Vibrio cholerae*. Infect. Immun. 67, 1393–1404. 10024587 10.1128/iai.67.3.1393-1404.1999 PMC96473

[B31] Gahlmann A. Moerner W. (2014). Exploring bacterial cell biology with single-molecule tracking and super-resolution imaging. Nature Rev. Microbiol. 12, 9–22. 10.1038/nrmicro3154 24336182 PMC3934628

[B32] Galán J. E. Lara-Tejero M. Marlovits T. C. Wagner S. (2014). Bacterial type III secretion systems: specialized nanomachines for protein delivery into target cells. Annu. Rev. Microbiol. 68, 415–438. 10.1146/annurev-micro-092412-155725 25002086 PMC4388319

[B33] Gaytán M. O. Martínez-Santos V. I. Soto E. González-Pedrajo B. (2016). Type three secretion system in attaching and effacing pathogens. Front. Cell. Infect. Microbiol. 6:129. 10.3389/fcimb.2016.00129 27818950 PMC5073101

[B34] Gerc A. J. Diepold A. Trunk K. Porter M. Rickman C. Armitage J. P.. (2015). Visualization of the *Serratia* type vi secretion system reveals unprovoked attacks and dynamic assembly. Cell Rep. 12, 2131–2142. 10.1016/j.celrep.2015.08.053 26387948 PMC4594159

[B35] Ghosh A. S. Young K. D. (2005). Helical disposition of proteins and lipopolysaccharide in the outer membrane of *Escherichia coli*. J. Bacteriol. 187, 1913–1922. 10.1128/JB.187.6.1913-1922.2005 15743937 PMC1064060

[B36] Gibbs K. A. Isaac D. D. Xu J. Hendrix R. W. Silhavy T. J. Theriot J. A. (2004). Complex spatial distribution and dynamics of an abundant *Escherichia coli* outer membrane protein, LamB. Mol. Microbiol. 53, 1771–1783. 10.1111/j.1365-2958.2004.04242.x 15341654

[B37] Gustafsson M. G. (2000). Surpassing the lateral resolution limit by a factor of two using structured illumination microscopy. J. Microsc. 198, 82–87. 10.1046/j.1365-2818.2000.00710.x 10810003

[B38] Gustafsson M. G. (2005). Nonlinear structured-illumination microscopy: wide-field fluorescence imaging with theoretically unlimited resolution. Proc. Natl. Acad. Sci. U.S.A. 102, 13081–13086. 10.1073/pnas.0406877102 16141335 PMC1201569

[B39] Hancock R. (1987). Role of porins in outer membrane permeability. J. Bacteriol. 169, 929. 10.1128/jb.169.3.929-933.1987 2434461 PMC211881

[B40] Hancock R. E. Reeves P. (1976). Lipopolysaccharide-deficient, bacteriophage-resistant mutants of *Escherichia coli* K-12. J. Bacteriol. 127, 98–108. 776951 10.1128/jb.127.1.98-108.1976 PMC233038

[B41] Heilemann M. Dedecker P. Hofkens J. Sauer M. (2009). Photoswitches: key molecules for subdiffraction-resolution fluorescence imaging and molecular quantification. Laser Photon. Rev. 3, 180–202. 10.1002/lpor.200810043

[B42] Heinz E. Stubenrauch C. J. Grinter R. Croft N. P. Purcell A. W. Strugnell R. A.. (2016). Conserved features in the structure, mechanism, and biogenesis of the inverse autotransporter protein family. Genome Biol. Evol. 8, 1690–1705. 10.1093/gbe/evw112 27190006 PMC4943183

[B43] Hell S. W. (2007). Far-field optical nanoscopy. Science 316, 1153–1158. 10.1126/science.1137395 17525330

[B44] Hell S. W. (2009). Microscopy and its focal switch. Nat. Methods 6, 24–32. 10.1038/nmeth.1291 19116611

[B45] Hell S. W. Wichmann J. (1994). Breaking the diffraction resolution limit by stimulated emission: stimulated-emission-depletion fluorescence microscopy. Opt. Lett. 19, 780–782. 10.1364/OL.19.000780 19844443

[B46] Ho B. T. Dong T. G. Mekalanos J. J. (2014). A view to a kill: the bacterial type VI secretion system. Cell Host Microbe 15, 9–21. 10.1016/j.chom.2013.11.008 24332978 PMC3936019

[B47] Hofmann M. Eggeling C. Jakobs S. Hell S. W. (2005). Breaking the diffraction barrier in fluorescence microscopy at low light intensities by using reversibly photoswitchable proteins. Proc. Natl. Acad. Sci. U.S.A. 102, 17565–17569. 10.1073/pnas.0506010102 16314572 PMC1308899

[B48] Hood R. D. Singh P. Hsu F. Güvener T. Carl M. A. Trinidad R. R.. (2010). A type VI secretion system of *Pseudomonas aeruginosa* targets a toxin to bacteria. Cell Host Microbe 7, 25–37. 10.1016/j.chom.2009.12.007 20114026 PMC2831478

[B49] Hu B. Lara-Tejero M. Kong Q. Galan J. E. Liu J. (2017). *In situ* molecular architecture of the *Salmonella* type III secretion machine. Cell 168, 1065–1074.e1010. 10.1016/j.cell.2017.02.022 28283062 PMC5393631

[B50] Hu B. Margolin W. Molineux I. J. Liu J. (2015). Structural remodeling of bacteriophage T4 and host membranes during infection initiation. Proc. Natl. Acad. Sci. U.S.A. 112, E4919–E4928. 10.1073/pnas.1501064112 26283379 PMC4568249

[B51] Huang B. Babcock H. Zhuang X. (2010). Breaking the diffraction barrier: super-resolution imaging of cells. Cell 143, 1047–1058. 10.1016/j.cell.2010.12.002 21168201 PMC3272504

[B52] Huang B. Bates M. Zhuang X. (2009). Super-resolution fluorescence microscopy. Annu. Rev. Biochem. 78, 993–1016. 10.1146/annurev.biochem.77.061906.092014 19489737 PMC2835776

[B53] Jain S. van Ulsen P. Benz I. Schmidt M. A. Fernandez R. Tommassen J.. (2006). Polar localization of the autotransporter family of large bacterial virulence proteins. J. Bacteriol. 188, 4841–4850. 10.1128/JB.00326-06 16788193 PMC1483012

[B54] Jakubowski S. J. Krishnamoorthy V. Christie P. J. (2003). *Agrobacterium tumefaciens* VirB6 protein participates in formation of VirB7 and VirB9 complexes required for type IV secretion. J. Bacteriol. 185, 2867–2878. 10.1128/JB.185.9.2867-2878.2003 12700266 PMC154386

[B55] Janakiraman A. Goldberg M. B. (2004). Evidence for polar positional information independent of cell division and nucleoid occlusion. Proc. Natl. Acad. Sci. U.S.A. 101, 835–840. 10.1073/pnas.0305747101 14715903 PMC321767

[B56] Journet L. Cascales E. (2016). The type VI secretion system in *Escherichia coli* and related species. EcoSal Plus 7:ESP-0009-2015. 10.1128/ecosalplus.ESP-0009-2015 27223818 PMC11575709

[B57] Judd P. K. Kumar R. B. Das A. (2005). The type IV secretion apparatus protein VirB6 of *Agrobacterium tumefaciens* localizes to a cell pole. Mol. Microbiol. 55, 115–124. 10.1111/j.1365-2958.2004.04378.x 15612921

[B58] Kamiyama D. Huang B. (2012). Development in the STORM. Dev. Cell 23, 1103–1110. 10.1016/j.devcel.2012.10.003 23237944 PMC3523271

[B59] Kato N. Ohta M. Kido N. Ito H. Naito S. Hasegawa T.. (1990). Crystallization of R-form lipopolysaccharides from *Salmonella minnesota* and *Escherichia coli*. J. Bacteriol. 172, 1516–1528. 10.1128/jb.172.3.1516-1528.1990 2407725 PMC208628

[B60] Kato N. Sugiyama T. Naito S. Arakawa Y. Ito H. Kido N.. (2000). Molecular structure of bacterial endotoxin (*Escherichia coli* Re lipopolysaccharide): implications for formation of a novel heterogeneous lattice structure. Mol. Microbiol. 36, 796–805. 10.1046/j.1365-2958.2000.01893.x 10844668

[B61] Kiss T. Morgan E. Nagy G. (2007). Contribution of SPI-4 genes to the virulence of *Salmonella enterica*. FEMS Microbiol. Lett. 275, 153–159. 10.1111/j.1574-6968.2007.00871.x 17711458

[B62] Kjærgaard K. Schembri M. A. Hasman H. Klemm P. (2000). Antigen 43 from *Escherichia coli* induces inter-and intraspecies cell aggregation and changes in colony morphology of *Pseudomonas fluorescens*. J. Bacteriol. 182, 4789–4796. 10.1128/JB.182.17.4789-4796.2000 10940019 PMC111355

[B63] Klar T. A. Hell S. W. (1999). Subdiffraction resolution in far-field fluorescence microscopy. Opt. Lett. 24, 954–956. 10.1364/OL.24.000954 18073907

[B64] Koch A. L. (1996). What size should a bacterium be? A question of scale. Annu. Rev. Microbiol. 50, 317–348. 10.1146/annurev.micro.50.1.317 8905083

[B65] Kudryashev M. Diepold A. Amstutz M. Armitage J. P. Stahlberg H. Cornelis G. R. (2015). *Yersinia enterocolitica* type III secretion injectisomes form regularly spaced clusters, which incorporate new machines upon activation. Mol. Microbiol. 95, 875–884. 10.1111/mmi.12908 25524451

[B66] Kumar R. B. Das A. (2002). Polar location and functional domains of the *Agrobacterium tumefaciens* DNA transfer protein VirD4. Mol. Microbiol. 43, 1523–1532. 10.1046/j.1365-2958.2002.02829.x 11952902

[B67] Lieberman J. A. Frost N. A. Hoppert M. Fernandes P. J. Vogt S. L. Raivio T. L.. (2012). Outer membrane targeting, ultrastructure, and single molecule localization of the enteropathogenic *Escherichia coli* type IV pilus secretin BfpB. J. Bacteriol. 194, 1646–1658. 10.1128/JB.06330-11 22247509 PMC3302462

[B68] Lindner A. B. Madden R. Demarez A. Stewart E. J. Taddei F. (2008). Asymmetric segregation of protein aggregates is associated with cellular aging and rejuvenation. Proc. Natl. Acad. Sci. U.S.A. 105, 3076–3081. 10.1073/pnas.0708931105 18287048 PMC2268587

[B69] Lippincott-Schwartz J. Patterson G. H. (2009). Photoactivatable fluorescent proteins for diffraction-limited and super-resolution imaging. Trends Cell Biol. 19, 555–565. 10.1016/j.tcb.2009.09.003 19836954 PMC3663713

[B70] Low H. H. Gubellini F. Rivera-Calzada A. Braun N. Connery S. Dujeancourt A.. (2014). Structure of a type IV secretion system. Nature 508, 550–553. 10.1038/nature13081 24670658 PMC3998870

[B71] Lu Q. Xu Y. Yao Q. Niu M. Shao F. (2015). A polar-localized iron-binding protein determines the polar targeting of *Burkholderia* BimA autotransporter and actin tail formation. Cell Microbiol. 17, 408–424. 10.1111/cmi.12376 25293534

[B72] Lybarger S. R. Johnson T. L. Gray M. D. Sikora A. E. Sandkvist M. (2009). Docking and assembly of the type II secretion complex of *Vibrio cholerae*. J. Bacteriol. 191, 3149–3161. 10.1128/JB.01701-08 19251862 PMC2681814

[B73] MacIntyre D. L. Miyata S. T. Kitaoka M. Pukatzki S. (2010). The *Vibrio cholerae* type VI secretion system displays antimicrobial properties. Proc. Natl. Acad. Sci. U.S.A. 107, 19520–19524. 10.1073/pnas.1012931107 20974937 PMC2984155

[B74] Manley S. Gillette J. M. Patterson G. H. Shroff H. Hess H. F. Betzig E.. (2008). High-density mapping of single-molecule trajectories with photoactivated localization microscopy. Nat. Methods 5, 155–157. 10.1038/nmeth.1176 18193054

[B75] Marsh J. W. Taylor R. K. (1998). Identification of the *Vibrio cholerae* type 4 prepilin peptidase required for cholera toxin secretion and pilus formation. Mol. Microbiol. 29, 1481–1492. 10.1046/j.1365-2958.1998.01031.x 9781884

[B76] Matsumoto K. Hara H. Fishov I. Mileykovskaya E. Norris V. (2015). The membrane: transertion as an organizing principle in membrane heterogeneity. Front. Microbiol. 6:572. 10.3389/fmicb.2015.00572 26124753 PMC4464175

[B77] Morgan J. K. Luedtke B. E. Shaw E. I. (2010). Polar localization of the *Coxiella burnetii* type IVB secretion system. FEMS Microbiol. Lett. 305, 177–183. 10.1111/j.1574-6968.2010.01926.x 20199576 PMC2908181

[B78] Mougous J. D. Cuff M. E. Raunser S. Shen A. Zhou M. Gifford C. A.. (2006). A virulence locus of *Pseudomonas aeruginosa* encodes a protein secretion apparatus. Science 312, 1526–1530. 10.1126/science.1128393 16763151 PMC2800167

[B79] Mühlradt P. F. Menzel J. Golecki J. R. Speth V. (1973). Outer membrane of *Salmonella*. Eur. J. Biochem. 35, 471–481. 4581267 10.1111/j.1432-1033.1973.tb02861.x

[B80] Murdoch S. L. Trunk K. English G. Fritsch M. J. Pourkarimi E. Coulthurst S. J. (2011). The opportunistic pathogen *Serratia marcescens* utilizes type VI secretion to target bacterial competitors. J. Bacteriol. 193, 6057–6069. 10.1128/JB.05671-11 21890705 PMC3194891

[B81] Noinaj N. Gumbart J. C. Buchanan S. K. (2017). The β-barrel assembly machinery in motion. Nat. Rev. Microbiol. 15, 197–204. 10.1038/nrmicro.2016.191 28216659 PMC5455337

[B82] Notti R. Q. Bhattacharya S. Lilic M. Stebbins C. E. (2015). A common assembly module in injectisome and flagellar type III secretion sorting platforms. Nat. Commun. 6:7125. 10.1038/ncomms8125 25994170 PMC4443714

[B83] Notti R. Q. Stebbins C. E. (2016). The structure and function of type III secretion systems. Microbiol. Spectr. 4:VMBF-0004-2015. 10.1128/microbiolspec.VMBF-0004-2015 26999392 PMC4804468

[B84] Oddershede L. Dreyer J. K. Grego S. Brown S. Berg-Sørensen K. (2002). The motion of a single molecule, the λ-receptor, in the bacterial outer membrane. Biophys. J. 83, 3152–3161. 10.1016/S0006-3495(02)75318-6 12496085 PMC1302393

[B85] Overbye L. J. Sandkvist M. Bagdasarian M. (1993). Genes required for extracellular secretion of enterotoxin are clustered in *Vibrio cholerae*. Gene 132, 101–106. 10.1016/0378-1119(93)90520-D 8406031

[B86] Patterson G. Davidson M. Manley S. Lippincott-Schwartz J. (2010). Superresolution imaging using single-molecule localization. Annu. Rev. Phys. Chem. 61, 345–367. 10.1146/annurev.physchem.012809.103444 20055680 PMC3658623

[B87] Peabody C. R. Chung Y. J. Yen M. R. Vidal-Ingigliardi D. Pugsley A. P. Saier M. H. (2003). Type II protein secretion and its relationship to bacterial type IV pili and archaeal flagella. Microbiology 149, 3051–3072. 10.1099/mic.0.26364-0 14600218

[B88] Plummer A. M. Fleming K. G. (2016). From chaperones to the membrane with a BAM! Trends Biochem. Sci. 41, 872–882. 10.1016/j.tibs.2016.06.005 27450425 PMC5420074

[B89] Pukatzki S. Ma A. T. Revel A. T. Sturtevant D. Mekalanos J. J. (2007). Type VI secretion system translocates a phage tail spike-like protein into target cells where it cross-links actin. Proc. Natl. Acad. Sci. U.S.A. 104, 15508–15513. 10.1073/pnas.0706532104 17873062 PMC2000545

[B90] Pukatzki S. Ma A. T. Sturtevant D. Krastins B. Sarracino D. Nelson W. C.. (2006). Identification of a conserved bacterial protein secretion system in *Vibrio cholerae* using the *Dictyostelium* host model system. Proc. Natl. Acad. Sci. U.S.A. 103, 1528–1533. 10.1073/pnas.0510322103 16432199 PMC1345711

[B91] Radics J. Konigsmaier L. Marlovits T. C. (2014). Structure of a pathogenic type 3 secretion system in action. Nat. Struct. Mol. Biol. 21, 82–87. 10.1038/nsmb.2722 24317488

[B92] Rassam P. Copeland N. A. Birkholz O. Tóth C. Chavent M. Duncan A. L.. (2015). Supramolecular assemblies underpin turnover of outer membrane proteins in bacteria. Nature 523, 333–336. 10.1038/nature14461 26061769 PMC4905513

[B93] Ritchie K. Lill Y. Sood C. Lee H. Zhang S. (2013). Single-molecule imaging in live bacteria cells. Phil. Trans. R. Soc. B 368:20120355. 10.1098/rstb.2012.0355 23267188 PMC3538437

[B94] Rothenberg E. Sepúlveda L. A. Skinner S. O. Zeng L. Selvin P. R. Golding I. (2011). Single-virus tracking reveals a spatial receptor-dependent search mechanism. Biophys. J. 100, 2875–2882. 10.1016/j.bpj.2011.05.014 21689520 PMC3123979

[B95] Rust M. J. Bates M. Zhuang X. (2006). Sub-diffraction-limit imaging by stochastic optical reconstruction microscopy (STORM). Nat. Methods 3, 793–796. 10.1038/nmeth929 16896339 PMC2700296

[B96] Samsudin F. Ortiz-Suarez M. L. Piggot T. J. Bond P. J. Khalid S. (2016). OmpA: a flexible clamp for bacterial cell wall attachment. Structure 24, 2227–2235. 10.1016/j.str.2016.10.009 27866852

[B97] Sandkvist M. Michel L. O. Hough L. P. Morales V. M. Bagdasarian M. Koomey M.. (1997). General secretion pathway (eps) genes required for toxin secretion and outer membrane biogenesis in *Vibrio cholerae*. J. Bacteriol. 179, 6994–7003. 10.1128/jb.179.22.6994-7003.1997 9371445 PMC179639

[B98] Schindler M. Osborn M. J. Koppel D. E. (1980). Lateral diffusion of lipopolysaccharide in the outer membrane of *Salmonella typhimurium*. Nature 285, 261–263. 10.1038/285261a0 6990276

[B99] Schirmer T. Keller T. A. Wang Y.-F. Rosenbusch J. P. (1995). Structural basis for sugar translocation through maltoporin channels at 3.1 angstrom resolution. Science 267:512 10.1126/science.7824948 7824948

[B100] Schlumberger M. C. Müller A. J. Ehrbar K. Winnen B. Duss I. Stecher B.. (2005). Real-time imaging of type III secretion: *Salmonella* SipA injection into host cells. Proc. Natl. Acad. Sci. U.S.A. 102, 12548–12553. 10.1073/pnas.0503407102 16107539 PMC1194920

[B101] Schwarz S. Singh P. Robertson J. D. LeRoux M. Skerrett S. J. Goodlett D. R.. (2014). VgrG-5 is a *Burkholderia* type VI secretion system-exported protein required for multinucleated giant cell formation and virulence. Infect. Immun. 82, 1445–1452. 10.1128/IAI.01368-13 24452686 PMC3993412

[B102] Schwarz S. West T. E. Boyer F. Chiang W.-C. Carl M. A. Hood R. D.. (2010). *Burkholderia* type VI secretion systems have distinct roles in eukaryotic and bacterial cell interactions. PLoS Pathog. 6:e1001068. 10.1371/journal.ppat.1001068 20865170 PMC2928800

[B103] Shrivastava R. Miller J. F. (2009). Virulence factor secretion and translocation by *Bordetella* species. Curr. Opin. Microbiol. 12, 88–93. 10.1016/j.mib.2009.01.001 19186097 PMC2703423

[B104] Sikora A. E. Zielke R. A. Lawrence D. A. Andrews P. C. Sandkvist M. (2011). Proteomic analysis of the *Vibrio cholerae* type II secretome reveals new proteins, including three related serine proteases. J. Biol. Chem. 286, 16555–16566. 10.1074/jbc.M110.211078 21385872 PMC3089498

[B105] Slusky J. S. (2017). Outer membrane protein design. Curr. Opin. Struct. Biol. 45, 45–52. 10.1016/j.sbi.2016.11.003 27894013 PMC5445000

[B106] Smit J. Nikaido H. (1978). Outer membrane of gram-negative bacteria. XVIII. Electron microscopic studies on porin insertion sites and growth of cell surface of *Salmonella typhimurium*. J. Bacteriol. 135, 687–702. 355240 10.1128/jb.135.2.687-702.1978 PMC222430

[B107] So E. C. Mattheis C. Tate E. W. Frankel G. Schroeder G. N. (2015). Creating a customized intracellular niche: subversion of host cell signaling by *Legionella* type IV secretion system effectors 1. Can. J. Microbiol. 61, 617–635. 10.1139/cjm-2015-0166 26059316

[B108] Spector J. Zakharov S. Lill Y. Sharma O. Cramer W. A. Ritchie K. (2010). Mobility of BtuB and OmpF in the *Escherichia coli* outer membrane: implications for dynamic formation of a translocon complex. Biophys. J. 99, 3880–3886. 10.1016/j.bpj.2010.10.029 21156129 PMC3000481

[B109] Spreter T. Yip C. K. Sanowar S. Andre I. Kimbrough T. G. Vuckovic M.. (2009). A conserved structural motif mediates formation of the periplasmic rings in the type III secretion system. Nat. Struct. Mol. Biol. 16, 468–476. 10.1038/nsmb.1603 19396170 PMC2860953

[B110] Thiem S. Kentner D. Sourjik V. (2007). Positioning of chemosensory clusters in *E. coli* and its relation to cell division. EMBO J. 26, 1615–1623. 10.1038/sj.emboj.7601610 17332753 PMC1829377

[B111] Thiem S. Sourjik V. (2008). Stochastic assembly of chemoreceptor clusters in *Escherichia coli*. Mol. Microbiol. 68, 1228–1236. 10.1111/j.1365-2958.2008.06227.x 18476921

[B112] Thomas S. Holland I. B. Schmitt L. (2014). The type 1 secretion pathway—the hemolysin system and beyond. Biochim. Biophys. Acta 1843, 1629–1641. 10.1016/j.bbamcr.2013.09.017 24129268

[B113] Toomre D. Bewersdorf J. (2010). A new wave of cellular imaging. Annu. Rev. Cell Dev. Biol. 26, 285–314. 10.1146/annurev-cellbio-100109-104048 20929313

[B114] Trokter M. Felisberto-Rodrigues C. Christie P. J. Waksman G. (2014). Recent advances in the structural and molecular biology of type IV secretion systems. Curr. Opin. Struct. Biol. 27, 16–23. 10.1016/j.sbi.2014.02.006 24709394 PMC4182333

[B115] Turnbull L. Toyofuku M. Hynen A. L. Kurosawa M. Pessi G. Petty N. K.. (2016). Explosive cell lysis as a mechanism for the biogenesis of bacterial membrane vesicles and biofilms. Nat Commun. 7:11220. 10.1038/ncomms11220 27075392 PMC4834629

[B116] Ursell T. S. Trepagnier E. H. Huang K. C. Theriot J. A. (2012). Analysis of surface protein expression reveals the growth pattern of the gram-negative outer membrane. PLoS Comput. Biol. 8:e1002680. 10.1371/journal.pcbi.1002680 23028278 PMC3459847

[B117] van Ulsen P. ur Rahman S. U. Jong W. S. Daleke-Schermerhorn M. H. Luirink J. (2014). Type V secretion: from biogenesis to biotechnology. Biochim. Biophys. Acta 1843, 1592–1611. 10.1016/j.bbamcr.2013.11.006 24269841

[B118] Verhoeven G. S. Dogterom M. den Blaauwen T. (2013). Absence of long-range diffusion of OmpA in *E. coli* is not caused by its peptidoglycan binding domain. BMC Microbiol. 13:66 10.1186/1471-2180-13-66 23522061 PMC3637615

[B119] Voth D. E. Heinzen R. A. (2007). Lounging in a lysosome: the intracellular lifestyle of *Coxiella burnetii*. Cell. Microbiol. 9, 829–840. 10.1111/j.1462-5822.2007.00901.x 17381428

[B120] Wang H. Wingreen N. S. Mukhopadhyay R. (2008). Self-organized periodicity of protein clusters in growing bacteria. Phys. Rev. Lett. 101:218101. 10.1103/PhysRevLett.101.218101 19113453 PMC12380459

[B121] Ward D. V. Draper O. Zupan J. R. Zambryski P. C. (2002). Peptide linkage mapping of the *Agrobacterium tumefaciens* vir-encoded type IV secretion system reveals protein subassemblies. Proc. Natl. Acad. Sci. U.S.A. 99, 11493–11500. 10.1073/pnas.172390299 12177441 PMC123284

[B122] Wong A. R. Pearson J. S. Bright M. D. Munera D. Robinson K. S. Lee S. F.. (2011). Enteropathogenic and enterohaemorrhagic *Escherichia coli*: even more subversive elements. Mol. Microbiol. 80, 1420–1438. 10.1111/j.1365-2958.2011.07661.x 21488979

[B123] Xie X. S. Choi P. J. Li G. W. Lee N. K. Lia G. (2008). Single-molecule approach to molecular biology in living bacterial cells. Annu. Rev. Biophys. 37, 417–444. 10.1146/annurev.biophys.37.092607.174640 18573089

[B124] Zoued A. Brunet Y. R. Durand E. Aschtgen M. S. Logger L. Douzi B.. (2014). Architecture and assembly of the Type VI secretion system. Biochim. Biophys. Acta 1843, 1664–1673. 10.1016/j.bbamcr.2014.03.018 24681160

[B125] Zoued A. Durand E. Brunet Y. R. Spinelli S. Douzi B. Guzzo M.. (2016). Priming and polymerization of a bacterial contractile tail structure. Nature 531, 59–63. 10.1038/nature17182 26909579

[B126] Zupan J. Muth T. R. Draper O. Zambryski P. (2000). The transfer of DNA from *Agrobacterium tumefaciens* into plants: a feast of fundamental insights. Plant J. 23, 11–28. 10.1046/j.1365-313x.2000.00808.x 10929098

